# Two new species of *Oxycera* (Diptera, Stratiomyidae) from Ningxia, China

**DOI:** 10.3897/zookeys.198.2624

**Published:** 2012-05-30

**Authors:** Yang Zai-Hua, Yu Jin-Yong, Yang Mao-Fa

**Affiliations:** 1 Guizhou Academy of Forestry, Guiyang 550005, P. R. China; 2 Guizhou Academy of Forestry, Guiyang 550005, P.R. China; 3 Institute of Entomology, Guizhou University, Guiyang 550025, P.R. China

**Keywords:** Diptera, Stratiomyidae, *Oxycera*, new species, Ningxia, China

## Abstract

Two new speices, *Oxycera rozkosnyi* **sp. n.** and *Oxycera ningxiaensis* **sp. n.**, are described from Liupanshan Nature Reserve, Ningxia Hui Autonomous Region, Northwest China. All essential diagnostic characters are figured and possible relationships of both taxa are briefly discussed, and a new key to species of *Oxycera* from China. The type specimens are deposited in the Institute of Entomology, Guizhou University, Guiyang (GUGC).

## Introduction

The genus *Oxycera* Meigen was erected by Meigen (1803) on the basis of the type species *Musca hypoleon* Linnaeus [= *Oxycera trilineata* (L).]. At present 94 species are described worldwide (Woodley 2001; Üstüner and Hasbenli 2004, 2007; Yang et al. 2008, 2009; Li et al. 2009; Zhang et al. 2009, 2010); Wang et al. 2010). The highest number of species is known from the Palaearctic Region (61 spp.), followed by the Oriental Region (16 spp.), 11 spp. were found in the Afrotropical Region and 8 spp. in the Nearctic. As for research on the genus *Oxycera* in China, Kertész (1914) first described three new species from Taiwan, following which Pleske (1925), Séguy (1934), and Lindner (1940) described four species. Yang and Nagatomi (1993) recorded 13 spp. (not including *Oxycera meigenii*) from China and recently 8 new species and a new country record have been published for China (Li et al. 2009; Wang et al. 2010; Yang et al. 2008, 2009; Zhang et al. 2009, 2010). In the present paper, two new Chinese species are described.

## Material and methods

External morphology was studied under a stereoscopic microscope, and measurements were made with an ocular micrometer. The genital segments of the examined specimens were macerated in 10% KOH and were preserved in glycerin for examination. All photographs were taken through a Canon 450D Camera, and were edited by Helicon Focus and Photoshop CS softwares. Illustrations of the specimens were made with a Nikon SMZ800 stereomicroscope and scanned with Canon CanoScan 5600F^+^, and then imported into Adobe Photoshop CS for labeling and plate composition.

Specimens examined in this study were collected in Ningxia Hui Autonomous Region, and are deposited in the Institute of Entomology, Guizhou University, Guiyang, Guizhou Province, P. R. China (GUGC). Morphological terminology follows Merz and Haenni (2000).

### Key to species of *Oxycera* Meigen from China

**Table:** 

1	Abdomen wholly black, at most with very narrow yellow or reddish yellow distal margin of tergite 5	2
–	Abdomen with more extensive yellow or yellow green markings, dorsolateral spots general protruding inward	12
2	Wing smoky brown with hyaline spots, or hyaline with smoky brown parts	3
–	Wing membrane completely hyaline or completely smoky brown tinged	5
3	Wing hyaline with a large smoky brown spot near apex	4
–	Wing smoky brown with a triangular hyaline spot below discal cell and another hyaline spot extending through anal and anterior cubital cells (Taiwan)	*Oxycera fenestrata* (Kertész, 1914)
4	Female frons black with a pair of small yellow spots (Kertész 1914: Fig. 34) (Taiwan), male unknown	*Oxycera apicalis* (Kertész, 1914)
–	Female frons black, with two pairs of small yellow spots, the upper spots larger than the lower (Wang et al. 2010: Figs. 1–2), male unknown	*Oxycera cuiae* (Wang et al., 2010)
5	Wing wholly smoky brown	*Oxycera ningxiaensis* sp. n.
–	Wing not wholly smoky brown, at least with hyaline spot	6
6	Female frons with a pair of longitudinal brick red vittae (Yang et al. 2009: Fig. 5), male head almost completely covered with dense hairs, thorax and lateral margin of abdomen with long and erect hairs (Yang et al. 2009: Fig. 1, 3)	*Oxycera qiana* Yang et al., 2009
–	Female frons with paired yellow spots or very small brick red spots, male head only with sparse hairs or partly bare, hairs on thorax and abdomen mainly appressed	7
7	Abdomen wholly black	8
–	Abdomen black, but posterior margin of tergite 5 yellow	10
8	Scutellum black with dark yellow posterior margin between spines (Li et al. 2009: Fig. 9–10)	*Oxycera liui* Li et al., 2009
–	Scutellum black without dark yellow posterior margin between spines	9
9	Thorax black, but posterodorsal margin of anepisternum yellow; female frons with a pair of yellow spots (Yang and Nagatomi 1993: Figs. 16, 13)	*Oxycera guangxiensis* Yang & Nagatomi, 1993
–	Thorax wholly black; female frons with 2 pairs of yellow spots (Yang et al. 2008: Fig. 12, 10)	*Oxycera guizhouensis* Yang et al., 2008
10	Female frons with a pair of yellow median spots (Yang and Nagatomi 1993: Fig. 8), male unknown	11
–	Female frons with 2 pairs of yellow spots (Lindner 1940: fig. 7), male unknown	*Oxycera quadripartita* (Lindner, 1940)
11	Scutum (except humeral and postalar calli) and pleura wholly black (Yang and Nagatomi 1993: fig. 11), male unknown	*Oxycera chikuni* Yang & Nagatomi, 1993
–	Scutum and pleura with yellow stripes or spots; male unknown	*Oxycera excellens* (Kertész, 1914)
12	Scutum without paired median longitudinal yellow vittae (sometimes scutum with 4 small and inconspicuous yellow spots)	13
–	Scutum with paired median longitudinal yellow or yellowish green vittae	18
13	Vein R_4_ present, body larger (about 7mm)	14
–	Vein R_4_ absent, body smaller (about 4mm)	17
14	Scutellum mainly black	15
–	Scutellum mainly yellow	16
15	Scutellum entirely black, (Zhang et al. 2010: Fig. 4); abdominal dorsum tergite 2–5 with green yellow lateral spots (Zhang et al. 2010: Fig. 5); female unknown	*Oxycera daliensis* Zhang et al., 2010
–	Scutellum black with dark yellow posterior margin and spines (except tips) (Zhang et al. 2009: Fig. 4); abdominal dorsum only with a pair of small lateral spots on tergite 4 (Zhang et al. 2009: Fig. 6); female frons with a pair of small dark yellow lateral spots, only post margin and spines dark yellow (Zhang et al. 2009: Fig. 2); male unknown	*Oxycera basalis* Zhang et al. 2009
16	Abdominal dorsum with 2 pairs of spots (Fig. 1), female frons with a pair of small yellow spots at ventral corner (Fig. 4)	*Oxycera rozkosnyi* sp. n.
–	Abdominal dorsum with a pair of large diagonal lateral spots on tergite 3 (Yang and Nagatomi 1993: Fig. 22, 24); female frons with a pair of large yellow longitudinal vittae (Yang and Nagatomi 1993: Fig. 20)	*Oxycera laniger* (Seguy, 1934)
17	Female scutum with 4 small and inconspicuous median yellow spots (Yang and Nagatomi 1993: Fig. 31); abdominal dorsum with two pairs of lateral yellow spots, a median yellow spot on tergite 2, and an apical yellow spot on tergite 5 (Yang and Nagatomi 1993: Fig. 32); male abdominal tergite 2 and anterior part of tergite 3 with a large transverse yellow band (Yang et al. 2008: Fig. 5)	*Oxycera lii* Yang & Nagatomi, 1993
–	Female scutum without median yellow spots, abdominal dorsum only with a narrow whitish yellow lateral margin from distal margin of tergite 3 to distal margin of tergite 4 and a small apical spot on tergite 5 (Yang et al. 2009: Fig. 17); male unknown	*Oxycera micronigra* Yang et al., 2009
18	Abdomen mainly green or yellow with a black pattern (Rozkošný 1983: Pl. 51, Fig. 2)	*Oxycera trilineata* (Linnaeus, 1767)
–	Abdomen mainly black with contrasting yellow margins or spots	19
19	Spines on scutellum slender and nearly horizontal, area beyond spines not protruding posteriorly	20
–	Spines on scutellum stout and vertical, area beyond spines large and protruding posteriorly (Yang and Nagatomi 1993: Figs. 75, 76)	*Oxycera vertipila* Yang & Nagatomi, 1993
20	Vittae on scutum not touching anterior margin and transverse suture (Li et al. 2009: Fig. 5)	*Oxycera flavimaculata* Li et al., 2009
–	Vittae on scutum reaching anterior margin and at least touching transverse suture	21
21	Length of body shorter than wing, vittae on scutum reaching suture (Yang and Nagatomi 1993, Fig. 3)	*Oxycera qinghensis* Yang & Nagatomi, 1993
–	Length of body longer than wing, vittae on scutum extending at least slightly beyond transverse suture	22
22	Scutum with a pair of longitudinal vittae reaching anterior and hind margin	23
–	Scutum with a pair of longitudinal vittae touching yellow humeral spot and just beyond transverse suture, but not touching hind margin	24
23	Body larger (about 6.0 mm); male tergite 3 with a pair of lateral yellow spots (Yang et al. 2009: Fig. 18), female tergite 3 with four yellow spots in a transverse row (Yang et al. 2009: Fig. 19); male aedeagal complex bipartite (Yang et al. 2009: Fig. 26)	*Oxycera signata* Brunetti, 1920
–	Body smaller (about 4.5 mm); abdomen with transverse yellow band on tergite 3 in both sexes (rarely divided into 3 spots in some females); male aedeagal complex tripartite (Yang and Nagatomi 1993: Fig. 58)	*Oxycera tangi* Lindner, 1940
24	Antenna yellowish brown; median process of male genital capsule with two rounded lobes (Rozkošný 1983: Pl. 46, Fig. 7)	*Oxycera meigenii* Staeger, 1844
–	Antenna black; median process of male genital capsule with two subpointed lobes (Yang and Nagatomi 1993: Fig. 47)	*Oxycera sinica* (Pleske, 1925)

## Taxonomy

### Oxycera ningxiaensis sp. n.

urn:lsid:zoobank.org:act:A45DC709-3450-40CE-BC15-8DF33352540E

http://species-id.net/wiki/Oxycera_ningxiaensis

Figures 1–6

#### Holotype.

♂, Ningxia Hui Autonomous Region, JingYuan County, Liupanshan natural reserves, Dongshanpo, 2100 m, N35^o^36.767, E106^o^16.189, 28.viii.2009, Z.-H. Yang leg.

#### Paratypes.

3♂♂, the same locality labels as the holotype, all in GUGC.

#### Diagnosis.

Dark species with brownish yellow postpronotal callus, scutellar spines, postalar callus and narrow upper margin of anepisternum. Body hairs black, R_4_ present, legs mostly dark to black but bases and tips of coxae, femora and tibiae yellow, tarsi black but 1–2 basal tarsomeres on mid and hind tarsus yellow.

#### Description.

**Figures 1–6.**
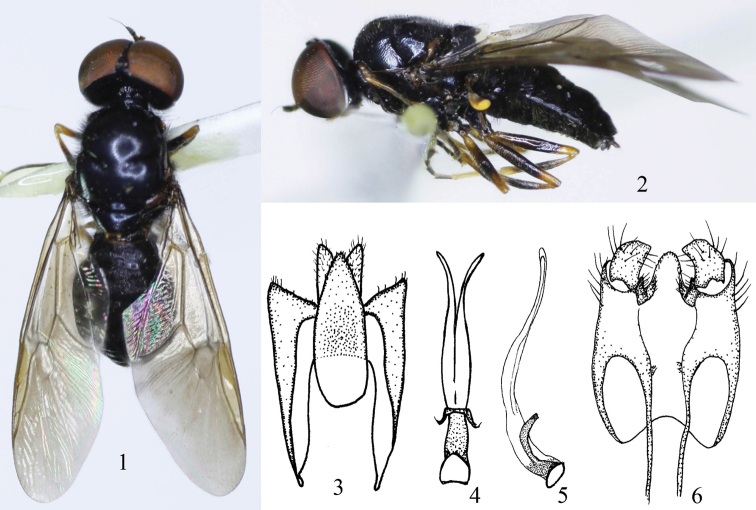
*Oxycera ningxiaensis* sp. n. holotype **1** Male, dorsal view **2** Male, lateral view **3** Proctiger, cerci and epandrium, ventral view **4–5** Aedeagal complex in dorsal and lateral view **6** Genital capsule, dorsal view.

Male (Figs 1–6). Length: body 4.8–5.2 mm, wing 4.3–5.0 mm. Head elliptical in frontal view, shining black, with black hairs; slightly broader than thorax, 1.4 times as long as high in profile and 0.8 times as high as broad. Vertex and ocellar tubercle black, both with black hairs. Ocelli and contiguous eyes brown, nearly bare, slightly darker in lower part, upper facets distinctly larger than lower. Frons shiny black; face black with some punctures and pale hairs, and a white lateral pollinose stripe on each side along eyes. Antenna dark brown to black, relative length of antennal scape, pedicel and flagellum (without arista) 3:5:10, relative width 7:9:10; arista about equal to length of rest of antenna. Occiput shiny black with some black hairs. Proboscis pale and palpus dark brown, both with some pale hairs.

Thorax (Figs 1, 2) mostly shining black, densely punctate and black haired; postpronotal callus, scutellar spines, postalar callus and narrow upper margin of anepisternum yellowish brown; length of scutellar spines only 1/4 as long as scutellum length. Wing black, stigma and veins darker than the mambrane, vein R_4_ present. Legs mostly dark brown to black, but each femur and tibia with yellow base and apex, mid and hind tarsi 1 yellow; legs wholly with short pale hairs. Halter yellow with yellowish brown base.

Abdomen (Figs. 1, 2) about as long as thorax, shining black, densely punctate, tergites 1–2 with dense black hairs, tergites 3–5 only with sparse pale hairs; similar hairs on venter. Male terminalia: epandrium trapezoidal (Fig. 3), its base narrower than tip, apical margin with sparse short hairs; proctiger elongate-oval, genital capsule with high medial process at hind margin (Fig. 6); aedeagal complex (Figs. 4, 5) bilobate, each lobe narrowed and pointed apically.

Female. Unknown.

#### Remarks.

**Figures 7–10.**
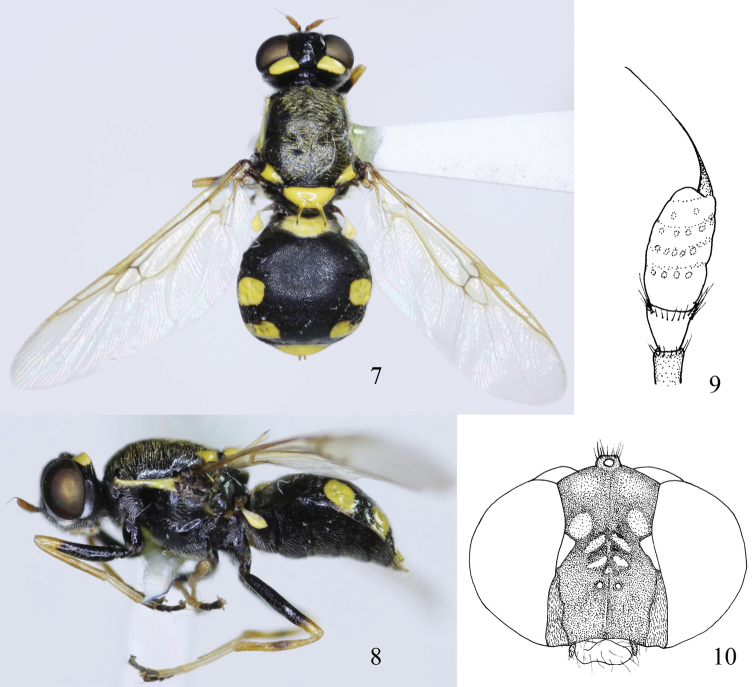
*Oxycera rozkosnyi* sp.n. holotype **7** Female, dorsal view **8** Female, lateral view **9** Antenna, inside **10** Head, frontal view.

This new species is very similar to *Oxycera qiana* Yang et al. 2009. Both are black but the new species is slender, with the scape and pedicel black, the male eyes are only sparsely haired or bare, and the male terminalia are species-specific (Figs 3–6). *Oxycera qiana* is stouter, with a somewhat paler scape and pedicel and densely haired eyes. The male terminalia are of quite different shape (cf. Figs 7–11 in Yang et al. 2009), i.e. the genital capsule is narrowed distally and without a medial process, and the aedeagal complex is trilobate.

#### Etymology.

The species is named after the type locality Ningxia in the Hui Autonomous Region.

#### Distribution.

China (Ningxia).

### Oxycera rozkosnyi sp. n.

urn:lsid:zoobank.org:act:0A25C653-526B-4A55-ACE0-A1F35B822FC0

http://species-id.net/wiki/Oxycera_rozkosnyi

Figures 7–10

#### Holotype.

♀, Ningxia Hui Autonomous Region, Jing Yuan County, Liupanshan natural reserves, Dongshanpo, 2100 m, N35^o^36.767; E106^o^16.189, 28.viii.2009, Z.-H. Yang leg.

#### Diagnosis.

Dark species without yellow stripes or spots on scutum (except postalar calli), eyes sparsely brown haired, legs mainly yellowish although femora mostly black. Abdomen with round yellow lateral markings on tergites 3 and 4 and a large central spot on tergite 1, and the posterior portion of tergite 5 yellow.

#### Description.

Male unknown.

Female (Figs 7–10). Length: body 6.3mm, wing 5.6 mm.

Head (Figs 7–8, 10) shining black with yellow pattern, 1.5 times as high as long in profile and 0.7–0.8 as high as broad in dorsal view. Frons with 3 pairs of medial pruinose yellow spots above antennae and a subtriangular yellow spot at eye margin on each side. Eyes sparsely short brown haired. Postocular rim with a oblong yellow spot on upper part and a pale subtriangular spot above postgena. Antenna (Fig. 9) yellowish brown, but scape and basal part of pedicel dark brown; relative lengths of antennal scape, pedicel and flagellum (without arista) 1:1.5:4, relative widths 5:7:9; arista about 0.9 times as long as rest of antenna. Face with white pollinose stripes along eye margin at each side. Hairs on head pale. Proboscis (Fig. 8) yellow, palpus dark brown, both pale haired.

Thorax (Figs. 7–8) shiny black. including postpronotal callus, scutum black, with whitish yellow hairs; postalar callus with a small subtriangular yellow anterior spot. Scutellum yellow, covered with sparse yellow hairs, spines yellow with dark tips; anepisternum with a narrow yellow stripe at upper margin from postalar callus to wing base; entire pleura with pale hairs. Legs: coxae and basal 4/5 of femora black, 3^r^^d^ to 5^th^ tarsomeres dark brown to black, rest of legs yellow to yellowish brown though tibiae slightly darkened at middle. Wing hyaline, veins pale yellow to brownish yellow, vein R_4_ present. Halter yellow with dark brown base.

Abdomen (Figs. 7–8) shining black with following yellow pattern (Fig. 7): tergite 1 with a large central spot, tergites 3 and 4 each with a pair of yellow lateral spots, tergite 5 with yellow posterior margin. Dorsum densely punctate and sparsely haired; venter entirely black, entire abdomen pale haired.

#### Remarks.

This new species is similar to *Oxycera dives* Loew, 1845 and *Oxycera locuples* Loew, 1857 known from Europe, but it may be separated from both by the missing dorsolateral stripes on the scutum and the large central spot on tergite 1. Lateral markings on tergite 2 are absent in the new species (and usually also in *Oxycera dives*) but distinct in *Oxycera locuples*. Using the most recent key to species of *Oxycera* from China (Zhang et al. 2010) the new species runs to couplet 9 (R_4_ present) but spines on the scutellum are not almost vertical.

#### Etymology.

The species is named in honor of Prof. Rudolf Rozkošný, a prominent Czech dipterist who contributed significantly to the knowledge of Palaearctic and Oriental Stratiomyidae.

#### Distribution.

China (Ningxia).

## Supplementary Material

XML Treatment for Oxycera ningxiaensis

XML Treatment for Oxycera rozkosnyi
